# The perspectives of bereaved family carers on dying at home: the study protocol of ‘unpacking the home: family carers’ reflections on dying at home

**DOI:** 10.1186/1472-684X-11-23

**Published:** 2012-11-22

**Authors:** Sheila Payne, Sarah Brearley, Christine Milligan, David Seamark, Carol Thomas, Xu Wang, Susan Blake, Mary Turner

**Affiliations:** 1 International Observatory on End of Life Care, Faculty of Health and Medicine, Lancaster University, Lancaster, Lancashire, LA1 4YT, United Kingdom; 2 Division of Health Research, Faculty of Health and Medicine, Lancaster University, Lancaster, Lancashire, LA1 4YT, United Kingdom; 3 Honiton Group Practice, Marlpits Lane, Honiton, Devon, EX14 2NY, United Kingdom; 4 Department of Psychology, Leeds Metropolitan University, Leeds, LS1 3HE, United Kingdom

**Keywords:** Informal caregivers, Palliative care, Home, Qualitative methods, Interviews, End of life

## Abstract

**Background:**

Recent end of life care policy prioritises patient choice over place of care and in particular promotes dying at home. This policy is predicated on the assumption that there are family carers able and willing to provide care for the dying person. Through the accounts of bereaved family members, the ‘Unpacking the home’ study aims to gain an in-depth understanding of ‘home’ and the issues faced by family members caring for a dying older person at home; it also aims to examine the way the home is transformed in the process of providing end of life care, and offer a critical analysis of policies that aim to increase home deaths. This paper presents the protocol for this study.

**Methods/design:**

A cross-sectional qualitative study has been designed to achieve the study aims. In-depth interviews will be conducted in the north and south of England with 50 bereaved family carers to elicit their accounts of witnessing the dying in the home of an older person (50+ years). All interviews will be subjected to thematic analysis, and narrative analysis will be undertaken on a subset of 30 interview transcripts. A final phase of integration and policy analysis will be conducted towards the end of the study. User involvement is integral to this study, with service users actively engaged at every stage.

**Discussion:**

This study will seek to take a qualitative approach by explicitly recognising that family carers are central to the experience of dying at home for older people, and they have needs that may be amenable to support and anticipatory planning. The strengths of this study, which include its interdisciplinary and participatory approach, and in-depth data collection and analysis methods, will be explored. The limitations and challenges of this research will also be considered. This study seeks to make recommendations that will ensure that family carers receive appropriate and adequate support in caring for their loved ones at the end of life.

## Background

Recent end of life care policy in the United Kingdom emphasises the importance of patient choice regarding place of care, and in particular promotes dying at home [1]. This policy is predicated on the assumption that there are family carers able and willing to provide care for the dying person (we use the National Institute for Clinical Excellence’s [2] broad definition of family carers as people with a close social and emotional bond, not just those related by kinship or marriage). Within Europe there are estimated to be 100 million family carers whose contribution to care often exceeds the financial expenditures of their countries on formal nursing services, although it is difficult to estimate exactly how many are engaged in caring for a person near the end of life [3]. Despite the important work that carers contribute, there is increasing evidence that they are often unprepared for the many demands they might face and that they experience considerable physical, psychological, social and financial challenges [4].

In the United Kingdom, 83.5% of all deaths occur in those over 65 years and evidence suggests that older people are ‘the disadvantaged dying’, with less access to health and social care services than younger people [5]. There is a divergence in reported preferences for dying at home (approximately 50-60%) [6], and actual deaths at home (which happen for less than 20%), although there are marked regional variations. The recent End of Life Care Strategy [1] puts emphasis on enabling patients to make choices about place of care and increasing home death rates. However, there is a growing critique that these policies fail to acknowledge the needs and preferences of older people [7], and that older people themselves may not regard home deaths as feasible or appropriate [8,9].

The availability of family carers able and willing to provide care at home is a key determinant in achieving a home death for cancer patients [9,10] and a major reason for admission to hospital is breakdown in family care. While this central role for carers is increasingly recognised, knowledge gaps remain about how to provide appropriate support to them during the dying phase [11]. When the home becomes the site of care for dying older people, the relocation of care work from institutional to domestic settings can create tensions between home and these types of work environments that can fundamentally challenge the meaning of home. Formal care workers, such as nurses and care assistants, need workspaces that are clean, hygienic and efficient for the purpose of delivering care [12]. This frequently requires the reorganisation of domestic space to accommodate the ‘paraphernalia of care’ [13], (for example, a hospital bed, commode, syringe driver, etc.). Whilst such artefacts are routinely available in institutional settings, these spaces can be organised to conceal some of the more disconcerting features in ways that cannot be easily achieved in domestic settings [14]. There has been only limited investigation of the ways in which the importation of artefacts associated with institutional care can affect the meaning attached to the domestic home [8,15]. Yet older people and their carers are unlikely to welcome the reordering of the home as a clinical work space, instead placing value on the home as a private, comfortable and aesthetically pleasing space that is imbued with personal memories and a sense of history and belonging [13].

The differing requirements of home and work for older people, informal and formal caregivers mean that the physical and symbolic meaning of the home must constantly be negotiated as both a site of care and of social and personal life. The significance of home as a social space, for example, points to why healthcare providers may encounter resistance from older people and their families in their attempts to reorganise domestic settings to accommodate the end of life care needs of the care-recipient [16] (for example the provision of Hospice at Home services). Hence the desire to improvise or subvert the logics of care-aids in order to retain a sense of home produces an ambiguity of place for both carer and care recipient – one that brings home and care into tension as the aesthetics of health care systems jostle against the aesthetics of home.

So while professional care within the home may be beneficial to the carer and care-recipient, they also transgress the social space and ‘normal’ domestic functioning of the home, creating a change in the meaning and sense of home. Work around end of life care and ‘place of death’ [8,9,17] suggests that whilst older people may initially prefer to be cared for by carers within the home, as levels of care needs intensify, the nature of home changes such that many would prefer to be cared for elsewhere. Any attempt to understand the implications and experiences of policies designed to support the home death for older people and their family carers thus brings into focus the complexity of the home both as a site of social interaction and personal meaning – and as a site of care that brings both the public and the private into tension [18]. These ‘felt’ changes in the home environment may have important embodied health and psychological effects for family carers.

The ‘Unpacking the home’ study was designed with the primary aim of gaining an in-depth understanding of ‘home’ and the issues faced by family members caring for a dying older person at home. Through the accounts of bereaved family carers this research will also examine the way the home is transformed in the process of providing end of life care, and offer a critical analysis of policies that aim to increase home deaths and how they impact on family carers during the period of care and into early bereavement. The specific objectives of the study are:

● To elicit the accounts of family carers about witnessing the dying in the home of an older person;

● To elicit views about the practical and other types of support that made this possible and their sources;

● To elicit views of deficits or gaps in support;

● To identify how the social and emotional ‘space’ of home is transformed during the process of care-giving, and how this is ‘felt’ in an embodied sense;

● To elicit perceptions of how the dying process and associated memories have impacted upon the use and feelings about ‘home’ during early bereavement; and

● To critically evaluate these carers’ accounts to identify the practical benefits and drawbacks of current policy, especially for family carers and dying older people.

## Methods/design

### Study design

This two-year study uses a participatory model of qualitative research, combining expertise from social gerontology, health geography, medical sociology, health psychology, primary care, nursing and end of life care to investigate the ways in which ‘dying at home’ is constructed, enacted and ‘felt’ in an embodied sense from the accounts of family carers. The study’s inductive approach is informed by the principles of grounded theory methods [19,20] and narrative analysis [21,22]. It also uses maximum variety sampling because a range of experiences is sought, rather than seeking to make generalisations based on population characteristics.

The study is being conducted in two sites in the North West (Lancashire and Cumbria) and South West (East Devon) of England. These regions have been selected as there are high proportions of older residents; they also enable identification of different levels of socioeconomic status (higher deprivation in Blackpool and Morecambe versus more affluence in East Devon), types of home ownership (owner-occupied versus rented), ethnic and cultural diversity, and health indices such as smoking rates (high in North West, low in South West).

### Setting and participants

The setting for this research is primary care, and participants are bereaved family carers recruited through GP practices. Researchers arrange meetings with GPs or other appropriate staff members (e.g. research nurses or practice managers) in each participating practice, to provide verbal and written information about the study and answer any questions. Practice staff then undertake database searches to identify family carers who meet all the study criteria, and information packs are posted to potential participants who respond directly to the research team if they are interested in taking part. Interviews take place in the participants’ homes.

### Inclusion criteria

● Family carers of older deceased people (aged 50 years +) from any cause of anticipated death;

● Death occurring in the home of the carer

● Two weeks minimum period of care prior to death

● Any age of adult carer (excluding children)

● Participants will be recruited at least 6 months but not more than 24 months following the death

### Exclusion criteria

● Sudden deaths e.g. stroke, MI

● Death in hospital or other institution

● Less than a two-week period of care at home prior to death

● Carers below age of 18

● Participants in early bereavement (less than 6 months) or more than 24 months

### Data collection

Data are collected through in-depth semi-structured interviews with bereaved carers. An interview schedule has been developed by the research team to elicit chronological narratives of care provision during the dying process, death and early period of bereavement. Interviews are digitally recorded and then fully transcribed. In addition, participants are invited to write their own accounts of their experiences of caring if they wish.

### Data analysis

Data analysis consists of two complementary analyses with a final phase of integration to offer insights from the findings and a critique of policies:

1. *Cross-sectional thematic analysis:* Using the principles of grounded theory, commonalities and differences are identified both within the individual accounts and across the two study sites. The analysis is influenced in an iterative way by the existing literature in order to maximize interpretative depth and ensure that the diversity of experiences for older people are fully addressed. An initial framework of thematic categories is applied to interview data drawing on the research objectives. A process of constant comparison is undertaken so that the early stages of analysis inform subsequent data collection and emergent issues will be pursued throughout the research process. The aim of the analysis is to produce insights relating to the research questions which are grounded in the experiences and understandings of the participants, and which are capable of theoretical or ‘logical’ generalization.

2. *Narrative analysis* of a subgroup of 30 transcripts (15 from each area) is being undertaken. The transcripts are selected to represent those carers who have experienced ‘positive’ and ‘negative’ home deaths to determine the extent to which these experiences influence their perceptions of home care, and their subsequent choices (such as selling their home). We are being guided by service users in defining what constitutes ‘positive’ and ‘negative’ experiences, recognising that delineation of these categories is complex. The analysis is informed by the methods described by Reissman [21] and Thomas [23]. We assess the quality of the analysis using a framework for establishing transferability and credibility in qualitative research [20]. During the whole process, the ‘lenses’ of social gerontology, health geography, medical sociology and end of life care with their disciplinary theories is drawn upon to increase the depth and utility of our analysis.

3. *Integration and policy analysis:* During the final phase we are exploring the extent to which findings from the thematic analysis, where the research questions ‘frame’ the analytical process, and the narrative analysis, where new issues raised by participants emerge, are congruent. We are using these findings to offer a critique of current end of life care policy and make recommendations to support family carers of older people. This stage involves engagement with policy makers and advocacy groups including Age UK, Carers UK and Help the Hospices.

### User involvement

Services users and bereaved carers have been involved in this study since its inception. Two individuals who have experience of caring for a dying family member at home have joined the project team, and contributed to the research in a variety of ways; for example, helping to create the interview guide and undertaking sections of data analysis to develop the thematic framework. In addition, three other bereaved carers took part in pilot interviews at the start of the study to develop and consolidate the interview guide.

### Ethical considerations

Ethical approval for the study was given by the National Research Ethics Service (NRES) Committee North West on 9 May 2011 (reference 11/NW/0203), and a substantial amendment to expand the inclusion criteria was approved on 18 May 2012. Written informed consent is being obtained from all study participants. The research team is mindful that the topic of this investigation is sensitive, and that a potentially vulnerable population is being recruited; for this reason ongoing feedback from service users is considered crucial in guiding the research process.

## Discussion

The overall aim of this study is to gain a profound understanding of the issues encountered by family members when caring for an older person dying at home; these issues are likely to include the care needs of the dying person, the support needs of family carers and the impact on and transformation of the home environment. The research takes a qualitative approach by explicitly recognising that family carers are central to the experience of dying at home for older people, and they have needs that may be amenable to support and anticipatory planning. The study has a number of strengths, limitations and potential challenges which will now be considered.

### Strengths

One of the greatest strengths of this study lies in its qualitative approach, which allows rich exploration of the specific area of older people dying at home, as experienced by bereaved carers. The inclusive, participatory design of the research, which combines expertise from diverse disciplinary and lay backgrounds, adds considerably to both the strength and the uniqueness of this research. Members of the interdisciplinary research team are all contributing their perspectives and interpretations of the study and its data, which ensures that meanings are fully captured, explored and understood. The inclusion of service users and bereaved family carers in the research team is particularly valuable, ensuring that the study remains ‘grounded’ in the real world of caring for a dying person at home.

The study uses the method of in-depth semi-structured interviews, which allows participants to ‘tell their stories’ in a coherent way, focusing on the elements of the story which are of particular significance to them, without the constraints that might be present in a more structured method of data collection. The option of constructing written accounts of their experiences offers participants further flexibility to provide information that they see as important.

### Limitations

This study has some limitations, which principally arise from the need to conduct this type of research with extreme sensitivity and ethical rigour. There are obvious difficulties in recruiting bereaved people into research studies, which are well documented in the literature [24]. In this study, different ways of recruiting participants were considered, and it was decided to recruit through GP practices, on the assumption that people who have died at home where the death was anticipated will be known to their GP. However, this meant that the research team relied heavily on the co-operation and good will of GP practices and were not themselves able to recruit participants directly. For ethical reasons it was decided that only bereaved carers who were registered with the same practice as the deceased person would be approached; this limited the number of potential participants in each practice. In order to achieve the target number of interviews, a large number of GP practices were approached and visited.

This research relies heavily on the recollections and accounts of bereaved carers rather than data from other sources, and it is possible that respondents’ bereavement might influence their retrospective accounts of their experiences, which may be very different from those of the people they cared for. However, in view of the inherent ethical and practical difficulties in interviewing dying patients, we have chosen to focus on carers for this study.

### Challenges

There are some specific challenges inherent in this study, perhaps the greatest of which is to ensure that its findings will directly influence health policy in relation to dying at home and thereby have a positive impact on care. The critical analysis of contemporary policy that is inherent within the study methodology needs to be translated into constructive recommendations for minimising the impact on family carers and better supporting them during the process of care and in the period of their bereavement.

## Conclusion

This study recognises that family carers are central to the experience of dying at home, and that they have needs that might be amenable to support and anticipatory planning. It examines the ways in which the home becomes transformed during the process of providing end of life care, and the impact of this on carers both at the time of the dying and into the period of early bereavement. It also offers a critical analysis of current policy to increase home deaths, and seeks to make recommendations that will ultimately ensure that family carers receive appropriate and adequate support in caring for their loved ones at the end of life.

## Competing interests

The authors declare that they have no competing interests.

## Authors’ contributions

SP designed the paper. SB, CM, DS, CT, XW, MT, SB took part in discussions. SP did the literature search. SP and MT led the writing and collation of the paper. All authors approved the final version of the paper.

## Pre-publication history

The pre-publication history for this paper can be accessed here:

http://www.biomedcentral.com/1472-684X/11/23/prepub
